# Declining Trend of Hepatitis A Seroepidemiology in Association with Improved Public Health and Economic Status of Thailand

**DOI:** 10.1371/journal.pone.0151304

**Published:** 2016-03-23

**Authors:** Pattaratida Sa-nguanmoo, Nawarat Posuwan, Preeyaporn Vichaiwattana, Viboonsak Vuthitanachot, Siriporn Saelao, Monthana Foonoi, Apinya Fakthongyoo, Jamorn Makaroon, Klaita Srisingh, Duangporn Asawarachun, Somchai Owatanapanich, Norra Wutthiratkowit, Kraisorn Tohtubtiang, Sompong Vongpunsawad, Pornsak Yoocharoen, Yong Poovorawan

**Affiliations:** 1 Center of Excellence in Clinical Virology, Department of Pediatrics, Faculty of Medicine, Chulalongkorn University, Pathum Wan, Bangkok, Thailand; 2 Chumphae Hospital, Chum Phae, Khon Kaen, Thailand; 3 Uttaradit Hospital, Mueang Uttaradit, Uttaradit, Thailand; 4 Laplae Hospital, Laplae, Uttaradit, Thailand; 5 Naresuan University Hospital, Mueang Phitsanulok, Phitsanulok, Thailand; 6 Phra Nakhon Si Ayutthaya Hospital, Phra Nakhon Si Ayutthaya, Thailand; 7 King Narai Hospital, Mueang Lop Buri, Lop Buri, Thailand; 8 Narathiwat Ratchanakarin Hospital, Mueang Narathiwat, Narathiwat, Thailand; 9 Trang Hospital, Mueang Trang, Trang, Thailand; 10 Bureau of Epidemiology, Department of Disease Control, Ministry of Public Health, Mueang Nonthaburi, Nonthaburi, Thailand; Kaohsiung Chang Gung Memorial Hospital, TAIWAN

## Abstract

Hepatitis A virus (HAV) is transmitted via the fecal-oral route from contaminated food or water. As part of the most recent survey of viral hepatitis burden in Thailand, we analyzed the current seroprevalence of HAV in the country and compared with data dating back to 1971. From March to October, 2014, a total of 4,260 individuals between one month and 71 years of age from different geographical regions (North = 961; Central = 1,125; Northeast = 1,109; South = 1,065) were screened for anti-HAV IgG antibody using an automated chemiluminescent microparticle immunoassay. Overall, 34.53% (1,471/4,260) possessed anti-HAV IgG antibody, and the age-standardized seroprevalence was 48.6%. Seroprevalence rates were 27.3% (North), 30.8% (Central), 33.8% (Northeast) and 45.8% (South) and were markedly lower than in the past studies especially among younger age groups. The overall trend showed an increase in the age by which 50% of the population were anti-HAV IgG antibody: 4.48 years (1971–1972), 6 (1976), 12.49 (1990), 36.02 (2004) and 42.03 (2014).This suggests that Thailand is transitioning from low to very low HAV endemicity. Lower prevalence of HAV correlated with improved healthcare system as measured by decreased infant mortality rate and improved national economy based on increased GDP per capita. The aging HAV immuno-naïve population may be rendered susceptible to potential HAV outbreaks similar to those in industrialized countries and may benefit from targeted vaccination of high-risk groups.

## Introduction

Hepatitis A virus (HAV) infection is estimated at 1.5 million individuals annually worldwide [1]. HAV belongs to the family *Picornaviridae* and the genus *Hepatovirus* [2]. It is a non-enveloped virus with a 7.5 kb single-stranded positive-sense RNA genome. It is classified into six genotypes, designated I-VI. Genotypes I, II and III have been identified in humans, while genotypes IV, V and VI are simian [3]. Despite genome diversity, all genotypes represent a single serotype [4]. The major transmission route for HAV is through consumption of contaminated food or water, although occasional transmission from person to person (through sexual intercourse or blood transfusion) has been reported [5–9]. High prevalence of HAV is often associated with poor hygiene, lack of sanitation and low socio-economic status [10].

In addition to improved hygiene and sanitation, the incidence of HAV infection can be substantially reduced using an inactivated HAV vaccine, which can effectively protect from all genotypes. Administration of two doses of HAV vaccine provides a long-term immunity from infection for up to 15 years [11,12]. Among the endemic regions of Africa and Asia, most individuals acquire immunity early in life [13]. Therefore, the prevalence of HAV infection in developing countries with low socioeconomic status remains high because infection occurs in early childhood and vaccination is often not feasible [14–15].

As a developing nation in Southeast Asia, Thailand has attained an upper- to middle-income economy in the past decade concommittant with continued improvement in sanitation and hygiene [16]. Consequently, the proportion of the population with immunity to HAV as measured by anti-HAV antibodies demonstrated a steady decline [17–18]. To assess the most current HAV seroprevalence in the general population in association with socio-economic factors, we evaluated serum samples obtained from individuals living in different geographical regions of Thailand for HAV antibodies and determined the correlates of immunity to national healthcare and economic parameters.

## Materials and Methods

This study was part of the overall research consortium to assess the status of viral hepatitis in the country by examining the seroprevalence of hepatitis A, B and C in Thailand and was approved by the Institutional Review Board of the Faculty of Medicine, Chulalongkorn University (IRB No. 419/56). Individuals were informed of the study objective and written consents were obtained from all participants or their parents. Basic demographic information (age, gender, and address) was also obtained. All samples were de-identified and anonymous.

### Study population

**Fig 1:**
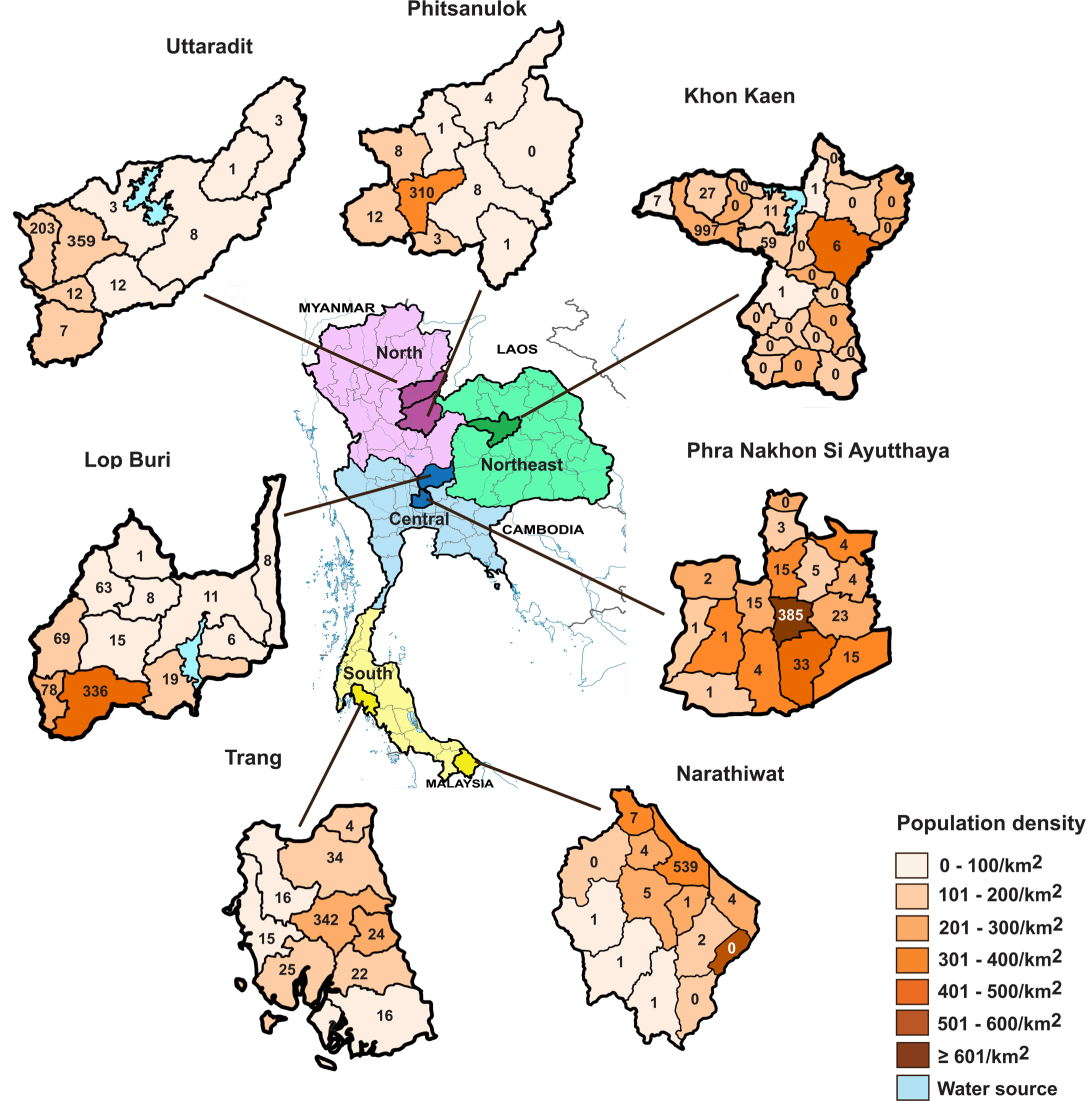
Map of Thailand and the domicile of study participants. Seven provinces from the four regions (North, violet; Northeast, green; Central, blue; and South, yellow) are shown. Districts within a given province are denoted in different colors depending on the population density (range 0 to ≥ 601 individuals/km^2^). Number of study participants from each district is indicated.

Blood samples were collected between March 1 and October 31, 2014 from patients during scheduled pediatric health check-up or outpatient clinic at hospitals in seven provinces representing four regions of Thailand (Fig 1). The provinces of Uttaradit and Phitsanulok represented the north, Lop Buri and Phra Nakhon Si Ayutthaya represented the central region, Khon Kaen represented the northeast, and Narathiwat and Trang represented the south. These provincial and district hospitals were chosen for their relatively good facilities and locations in generally comparable population densities. Samples were obtained from 4,260 individuals (1,792 males and 2,468 females; North = 961, Central = 1,125, Northeast = 1,109, and South = 1,065) (Table 1). The inclusion criteria were Thais residing in their respective provinces age between 1 month and 71 years of age. Exclusion criteria were immunological or hematologic disorder (e.g. HIV-positive status and hemophilia), malignancy, history of chronic diseases (e.g. heart and liver diseases), and congenital defects. Individuals who were hospitalized for longer than one month during the past year, undergoing steroid treatment, immunosuppressive drugs or chemotherapy were also excluded. Blood samples were separated into serum within 24 hours, transported to the Center of Excellence in Clinical Virology at King Chulalongkorn Memorial Hospital, and stored at -20°C until tested.

**Table 1:** Regions and demographics of individuals assessed for immunity to HAV.

Region	Province	Hospital name	District	Participants	Male	Female	Age range	Mean age ± SD (yr)
North	Uttaradit	Uttaradit Hospital	Mueang Uttaradit	614	268	346	6 mo–62 yr	22.9 ± 17.9
(n = 961)		Laplae Hospital	Laplae					
	Phitsanulok	Naresuan University Hospital	Mueang Phitsanulok	347	126	221	5 mo–68 yr	27.8 ± 18.7
Center	Lop Buri	King Narai Hospital	Khao Sam Yot	614	299	315	7 mo–71 yr	26.5 ± 18.4
(n = 1,125)	Phra Nakhon Si Ayutthaya	Phra Nakhon Si Ayutthaya Hospital	Phra Nakhon Si Ayutthaya	511	209	302	6 mo–60 yr	26.3 ± 17.7
Northeast	Khon Kaen	Chumphae Hospital	Chumphae	1,109	526	583	6 mo–61 yr	25.4 ± 17.7
(n = 1,109)								
South	Narathiwat	Narathiwat Ratchanakarin Hospital	Mueang Narathiwat	565	157	408	7 mo–60 yr	31.0 ± 16.9
(n = 1,065)	Trang	Trang Hospital	Mueang Trang	500	207	293	1 mo–62 yr	25.1 ± 25.1
TOTAL				4,260	1,792	2,468	1 mo–71 yr	26.5 ± 18.2

### Serological assay

Anti-HAV IgG was determined using the automated chemiluminescent microparticle immunoassay on the ARCHITECT *i*2000SR instrument (ARCHITECT HAVab-IgG, Abbott, Wiesbaden, Germany) according to the manufacturer’s instructions. The sensitivity and specificity of the test were > 98% and > 99.17%, respectively. Samples with the signal-to-cut-off (S/CO) ratio of > 1.00 were considered HAV antibody-positive by the automated assay, while samples with S/CO ratio < 1.00 were considered negative.

### Comparison of anti-HAV seroprevalence

Anti-HAV IgG prevalence obtained from this study was compared to the HAV seroprevalence data from past studies (Table 2). Briefly, the studies conducted in central region of Thailand in 1971–1972 and 1976 surveyed the urban and rural regions, respectively [16]. The 1991 study was conducted in the predominantly rural central region [17], while the 2007 study surveyed all four regions of the country similar to this study [18].

**Table 2:** Previous studies of anti-HAV IgG prevalence in Thailand.

Previous studies conducted	Year	Sample size	Location	% overall anti-HAV IgG positive
Burke *et al*. (1981) [16]	1971 to 1972	308	Huay Khwang, Bangkok	86.4
Burke *et al*. (1981) [16]	1976	206	Ban Tablan, Prachin Buri	59.7
Poovorawan *et al*. (1991) [17]	1990	364	Pong Nam Ron, Chanthaburi	64.5
		236	Bo Thong, Chon Buri	
Chatproedprai et al. (2007) [18]	2004	1,336	Chiang Rai	27.35
		985	Udon Thani	
		846	Nakhon Si Thammarat	
		830	Chon Buri	

To calculate the approximate age group with 50% detectable anti-HAV IgG, a line chart indicating the percentage of anti-HAV IgG seropositivity and age was constructed. The 50^th^ percentile of the anti-HAV-IgG seropositive rates allowed the determination of the approximate age group at which half of the population was HAV seropositive. Subsequently, a linear equation (y = mx + c) was derived from the chart trend line of each study, which gave a modifying factor used to allow the extrapolation of a more precise mean age with standard deviation (SD) at which 50% of the population possessed anti-HAV IgG antibodies.

### Economic and national health data

To determine the relationship between socio-economic status, health and HAV seroprevalence, the population of Thailand and other factors were considered. Gross domestic product (GDP) per capita in U.S. dollars [19] represented an economic indicator reflective of the standard of living. Infant mortality rate (defined as the number of infant deaths before one year of age per 1,000 live births in a given year) was used as an indicator for the quality of national healthcare [20]. The HAV annual morbidity rate per 100,000 individuals and location-specific HAV outbreak data were derived from the Bureau of Epidemiology, Department of Disease Control, Thai Ministry of Public Health [21].

### Statistical analysis

Statistical analysis (mean, SD and 95% CI) was performed using SPSS version 17.0 (SPSS Inc., Chicago, IL).

## Results

### Seroprevalence of anti-HAV IgG

Of the 4,260 persons sampled in this study, there were more women than men (57.9% versus 42.1%, respectively). The distribution of males and females were approximately equal for persons ≤ 14 years, but there were substantially more females than males ≥ 15 years (S1 Table).

**Fig 2:**
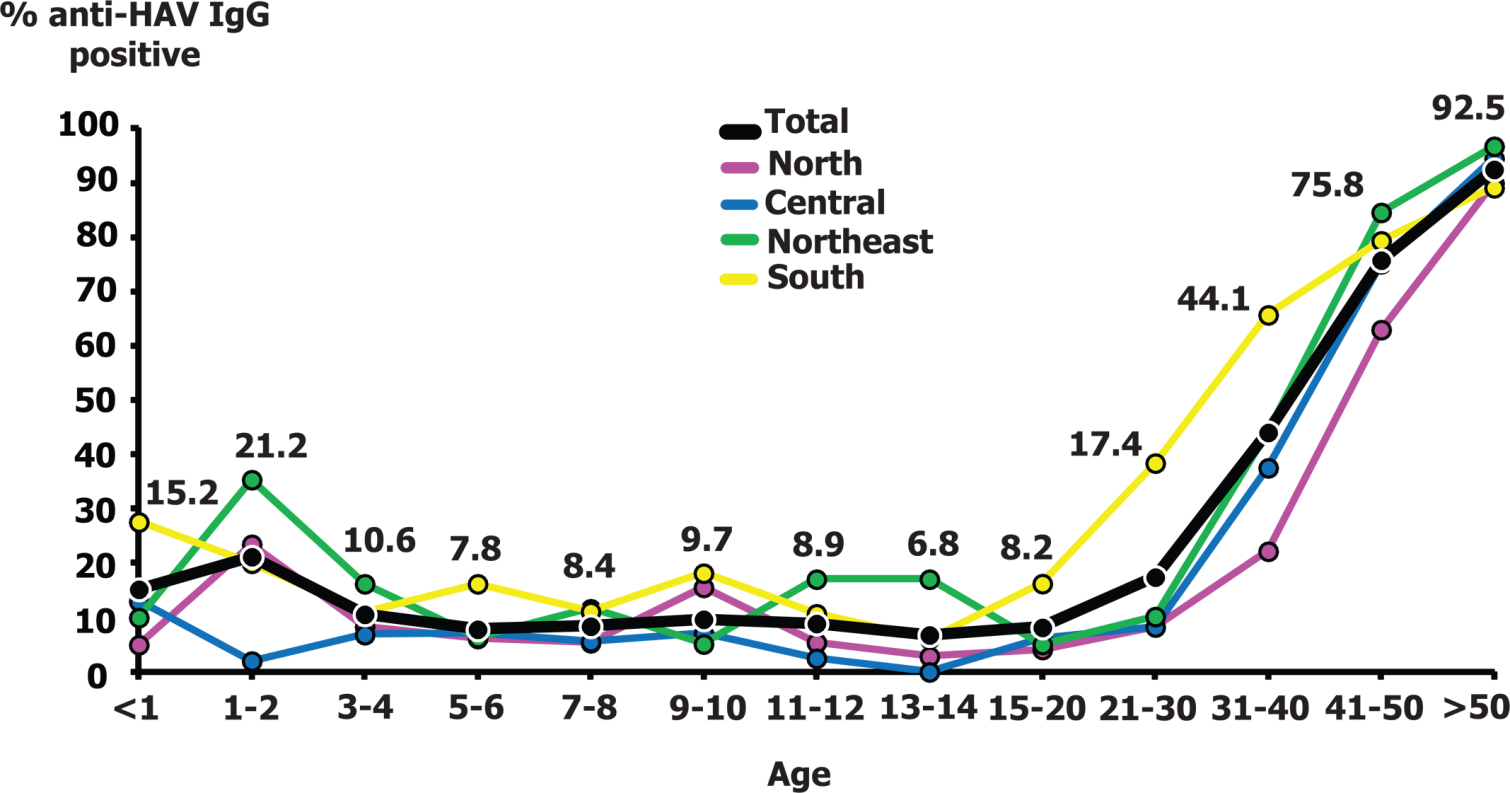
The frequency of anti-HAV IgG positivity in each age group. Numbers above the line charts indicate the total seroprevalence for each age group.

Seroprevalence progressively increased with age and was highest in individuals > 50 years (92.5%) in all regions (96.8% Northeast, 94.6% Central, 90.0% North, and 89.2% South). Regionally, we found the lowest anti-HAV IgG prevalence in the North (27.3%), while the highest was in the South (45.8%) especially among individuals 15–40 year-olds (Fig 2). The age-standardized anti-HAV IgG positive rate was 48.6% (Table 3).

**Table 3:** Expected number of age-specific anti-HAV antibody-positive Thais based on the anti-HAV IgG-seropositive rates in 2014.

Age group	Population	% anti-HAV positive rate	Anticipated anti-HAV IgG seropositive individuals
< 1	685,829	15.2	104,246
1–2	1,522,482	21.2	322,766
3–4	1,527,526	10.6	161,917
5–6	1,552,929	7.84	121,749
7–8	1,586,457	8.4	133,262
9–10	1,610,751	9.69	156,081
11–12	1,579,273	8.9	140,555
13–14	1,634,052	6.8	111,115
15–20	5,615,735	8.19	459,928
21–30	8,384,422	17.4	1,462,243
31–40	10,346,437	44.1	4,561,744
41–50	10,465,811	75.8	7,934,131
> 50	16,484,653	92.5	15,253,249
Total	62,996,357	48.6*	30,600,220

* Calculated from anticipated seropositive individuals divided by the population.

### Trends of anti-HAV IgG positivity from 1971 to 2014

**Fig 3:**
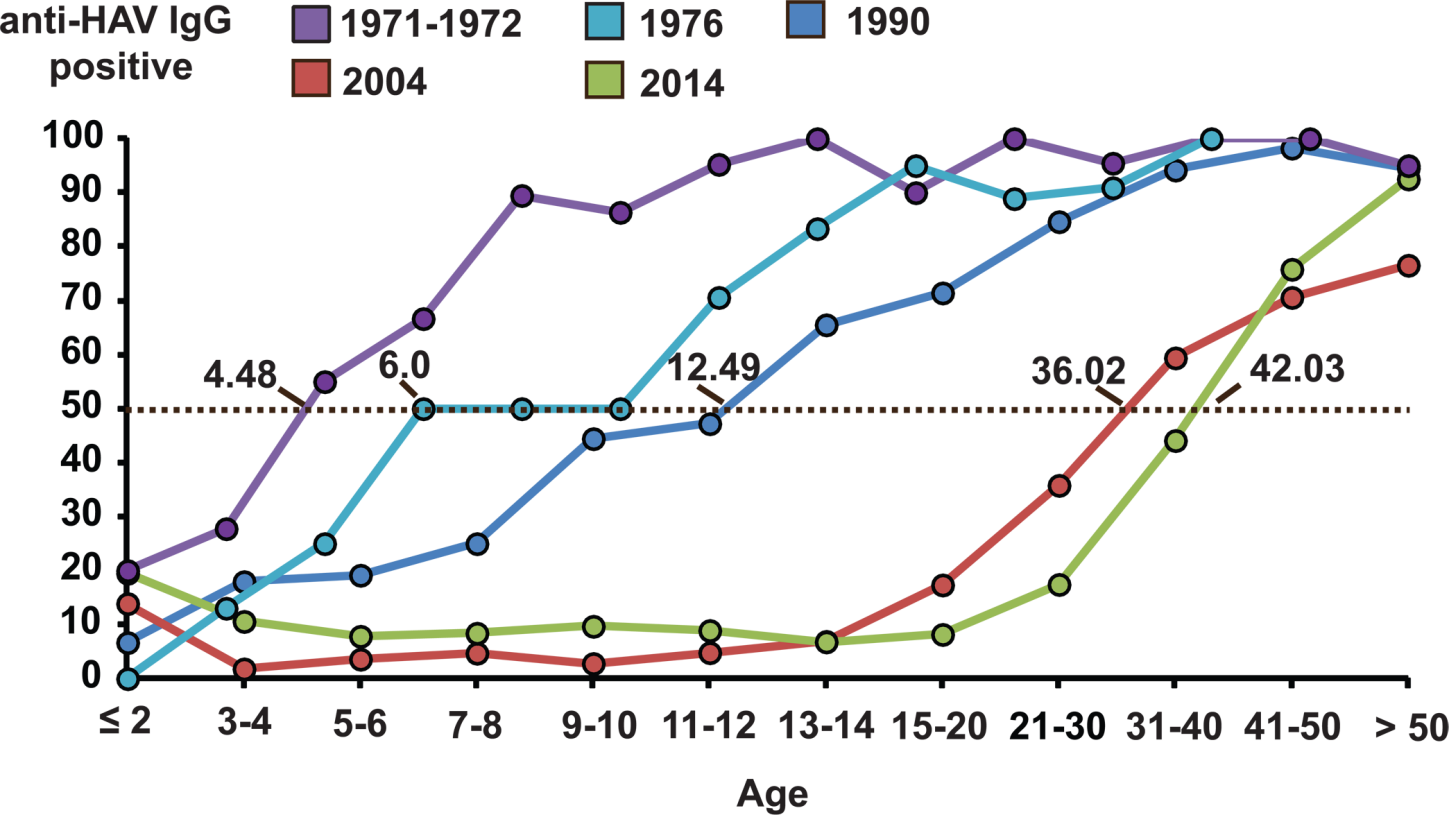
Comparison of anti-HAV IgG positivity from 1971 to 2014. Seroprevalence data from this and other studies were plotted as line charts. The dotted line denotes 50% anti-HAV IgG positivity. Intersection with the seroprevalence curve indicates the mean age at which 50% of the individuals in the population possessed anti-HAV IgG (denoted by the numbers on the line graphs) [16–18].

Seroprevalence of anti-HAV IgG in each age group was compared to age-specific prevalence of antibody to HAV from 4 previous studies (1971–1972, 1976, 1990 and 2004), which utilized similar immunoassay (Fig 3). Although studies conducted prior to the year 2000 only surveyed individuals residing in the central region of the country, serosurvey conducted in 2004 involved provinces spanning all four geographical regions and represented the most comprehensive seroprevalence similar to this study. Prior to 1990, nearly all young adults possessed anti-HAV antibody. This general pattern changed beginning in 2004 when most young adults were likely to be seronegative for HAV. To better characterize the delayed HAV seroconversion over the past 45 years, we determined the average age at which roughly half of the individuals in the population possessed anti-HAV antibody. Analysis showed that the HAV seropositive mean age progressively increased from ~4.5 years in the early 1970’s to ~42 years in 2014.

### Healthcare system and economic status correlated with changing HAV burden

**Fig 4:**
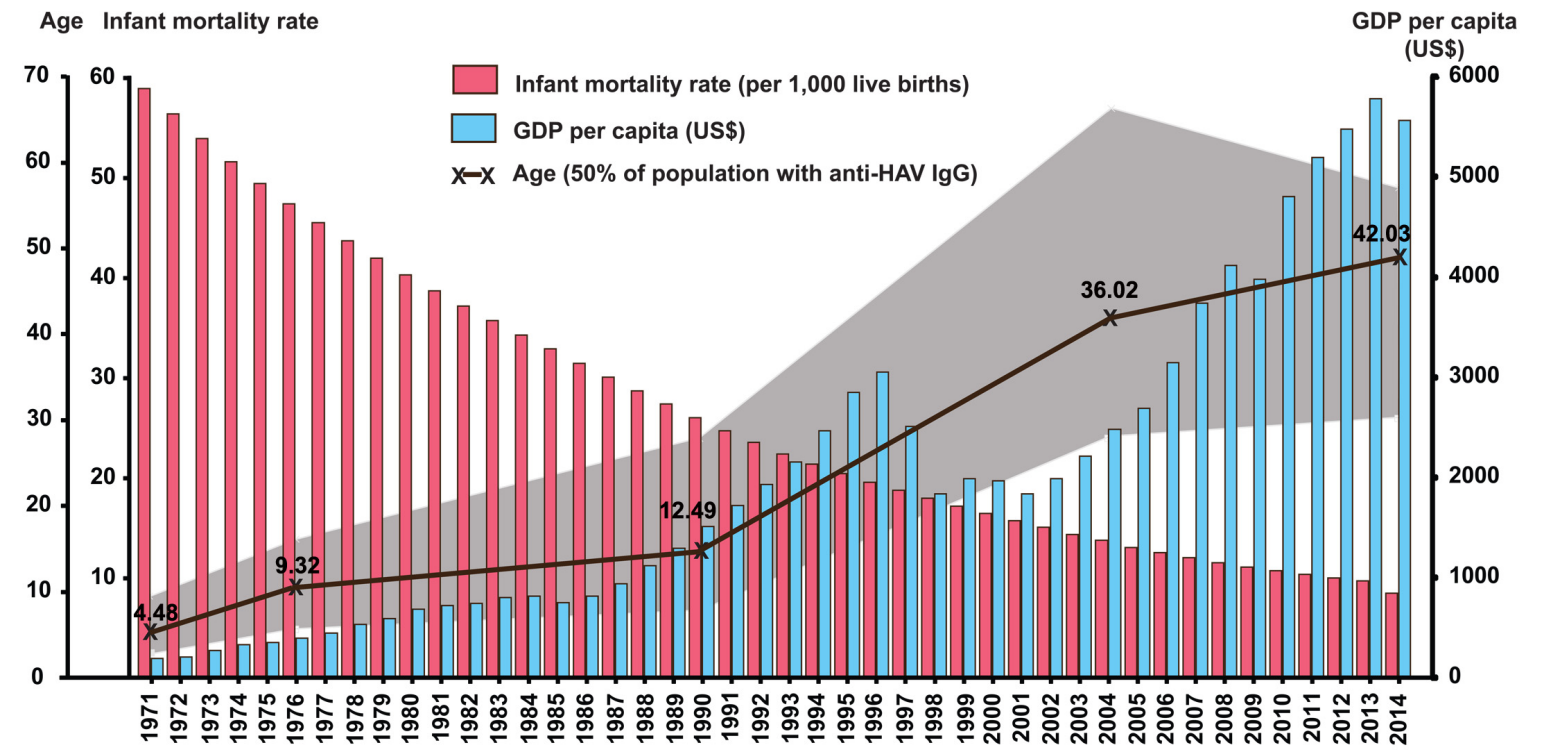
Correlation between infant mortality rate (per 1,000 live births), GDP, and age at which 50% of the population possessed anti-HAV antibody. Infant mortality rates (pink graphs), GDP per capita (blue graphs), and the mean age at 50% anti-HAV IgG positivity in the population (black line) are indicated. Half of the population around the mean age is denoted in gray areas.

**Fig 5:**
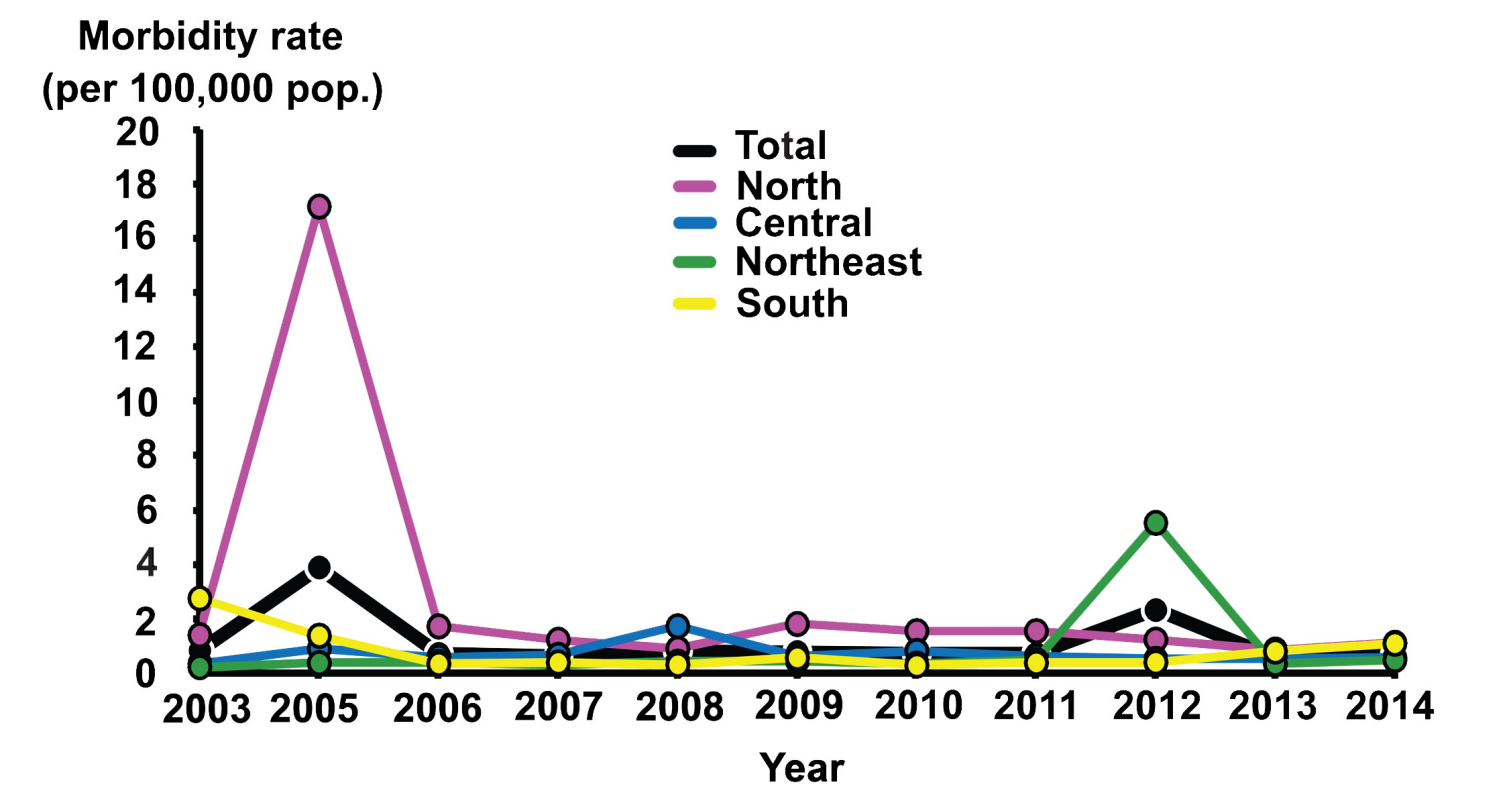
Total hepatitis A infection cases and morbidity rates in Thailand. Data available from 2003 to 2014 (excluding 2004) from the Bureau of Epidemiology, Thai Ministry of Public Health, are presented as line charts. Rates are indicated per 100,000 persons.

Since provincial and regional socio-economic data were not available for correlation with the seroprevalence finding, we used the available national data to analyze for potential association. While seroprevalence of HAV declined over the past 45 years, the infant mortality rate also decreased from ~60 per 1,000 to ~10 per 1,000 live births per year (Fig 4). Meanwhile, the GDP per capita steadily increased over the same period, suggesting an overall improved national economic status. The morbidity rates (per 100,000 individuals) associated with HAV infection available beginning in 2003 from the Bureau of Epidemiology, Thai Ministry of Public Health, indicated that between 2003 and 2014, HAV morbidity rate was less than 1 per 100,000 persons annually (Fig 5). Death associated with HAV infection is extremely rare, with the average mortality rate less than 0 per 100,000 persons annually during this period [21].

**Fig 6:**
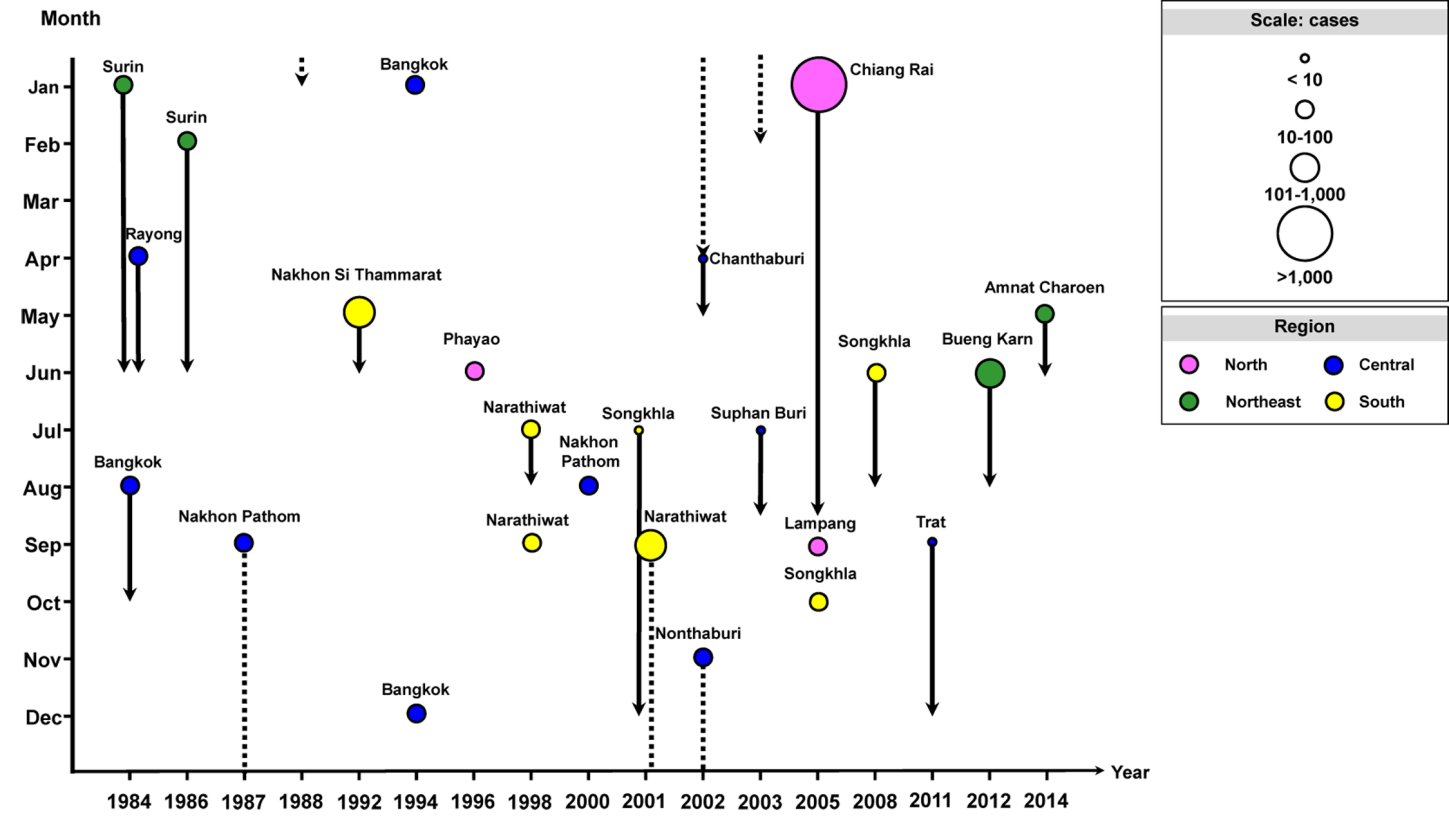
Documented hepatitis A outbreaks in Thailand from 1984 to 2014. Circles denote outbreak events in different regions (North, purple; Central, blue; Northeast, green; South, yellow). Circle sizes correspond to the number of individuals affected. Outbreak in a calendar year is denoted as solid lines; those that continued to the following year are denoted in dashed lines. Line length corresponds to the recorded outbreak period.

There were two recorded HAV outbreaks in the northern province of Chiang Rai and the northeastern province of Bueng Karn, which resulted in the spike in morbidity in 2005 (3.98) and 2012 (2.32). Epidemiological data also showed documented sporadic incidence of HAV outbreaks of varying scale over the past 30 years, which occurred in various provinces in Thailand (Fig 6).

## Discussion

A study published 40 years ago found that most Thais were infected with HAV before reaching adulthood [17]. Our study revealed that as the healthcare and economic status of Thailand improved, the prevalence of HAV antibodies in the population steadily declined. Although some middle-income countries in Southeast Asia represent low endemicity region as defined by the World Health Organization (≥ 50% of the population immune by age 30) [22–24], these most current seroprevalence data suggest that Thailand is transitioning to a very low HAV endemicity (defined by <50% having immunity by age 30 years).

Presently, most children and young adults are unlikely to possess immunity to HAV due to the observed low anti-HAV seroprevalence in the 3–20 year age groups (6.8–10.6%). Consequently, they are expected to be more susceptible to HAV infection should it occur later in life. Due to placental antibody transfer from mother to child, we continued to find a slight increase in the anti-HAV seroprevalence in children age < 1 year old. This passive antibody transfer will likely convey protection should infants be exposed to HAV early in life, but this is transient as the majority of acquired maternal antibodies declined with age [25, 26]. Consistent with previous sero-surveillance data, most individuals above 40 years possessed HAV antibodies, which may be attributed to the region’s lower standard of living and hygiene conditions in the first half of the century as well as higher exposure to HAV during childhood. Although under-reporting may have contributed to the very low morbidity and mortality rates for hepatitis A infection in Thailand, susceptibility to local HAV outbreaks in 2005 and 2012 (Figs 5 and 6) is consistent with the available scientific evidence that HAV is not highly endemic in this region. Nevertheless, morbidity rates can increase significantly in such instance as the majority of patients were adults.

The trend of lower HAV infection rates in recent years is underscored by the steady increase in the mean age with 50% anti-HAV IgG seropositivity in the population. The most significant change occurred between 1990 and 2004, from 12.49 years to 36.02 years. As uninfected children become adults, the HAV seroprevalence is predicted to further decline, which would shift the seroprevalence curved steadily to the right. The shift in the mean age in which 50% of the population possessed anti-HAV IgG was associated with several indicators, including higher socio-economic status as measured by the declining infant mortality rate, improved sanitation facilities and hygiene practices, and increased GDP per capita to support better living standards. It is envisioned that future seroprevalence will continue to decline due to lower HAV endemicity, although this will render the population susceptible to occasional outbreaks as seen in very low endemic and developed countries [27–29].

HAV infection is vaccine-preventable. However, vaccination is not universally implemented in many underdeveloped and developing countries because infection often through contaminated water and food is commonly asymptomatic and occurs at an early age, thus preventing symptomatic infection as adults. As sanitation and living conditions improved, limited exposure to HAV in children over time leads to susceptibility in adulthood. In developed countries with low rates of HAV infection, increased mobidity and mortality particularly in the unvaccinated elderly have been reported [30]. Since the hepatitis A vaccine is currently not included into the national Expanded Program of Immunization (EPI), the shift in seroprevalence trends we observed in this study are not due to vaccine implementation. Although the efficacy of HAV vaccination can lead to the prevention of infection in up to 94–100%, cost-benefit analysis does not appear to justify its widespread use in the country [31]. In low to very low endemic countries, targeted vaccination of high-risk groups has been recommended [32], which may prevent complications associated with hospitalization, morbidity and mortality [30].

There were several limitations to this study. More females than males in this hospital-based study may reflect the fact that women were more more likely to seek medical care when needed compare to men. Although questionnaires were administered to participants to gather socio-economic data, incomplete reporting of information prevented analyzable classification of socio-economic status that would have been useful. The observed fluctuation in the seroprevalence may be subjected to the differences in the cohort sampled over the years. In addition, declined HAV antibody titers in older individuals may also account for varying seroprevalence rates and that the specificity of immunological assay may underdetect individuals who have low anti-HAV antibodies. We also cannot determine if detectable HAV antibody in the serum resulted from past or active infection, although it is unlikely from immunization. Nevertheless, this most current HAV seroprevalence survey should assist in formulating policy and possible consideration for HAV vaccination in susceptible age groups to further facilitate the control and prevention of HAV.

## Supporting Information

S1 Table Distribution of anti-HAV IgG seropositivity in the study population. (DOCX)
