# Supplementary material for: Declining Trend of Hepatitis A Seroepidemiology in Association with Improved Public Health and Economic Status of Thailand
Source: PLoS One. 2016 Mar 23;11(3):e0151304. doi: 10.1371/journal.pone.0151304 (PMC4805277; doi:10.1371/journal.pone.0151304)
Supplement: S1 Table — (DOCX) [file pone.0151304.s001.docx]

**S1 Table. Distribution of anti-HAV IgG seropositivity in the study population.**

|  | **National** |  |  |  |  | **Regional** |  |  |  |  |  |  |  |
| --- | --- | --- | --- | --- | --- | --- | --- | --- | --- | --- | --- | --- | --- |
| **Age** | **Total** | **Male** | **Female** | **Positive** | **(%)** | **Central** | **(%)** | **Northeast** | **(%)** | **North** | **(%)** | **South** | **(%)** |
| <1 | 92 | 59 | 33 | 14 | 15.2 | 3/23 | 13.0 | 2/20 | 10.0 | 1/20 | 5.0 | 8/29 | 27.6 |
| 1-2 | 236 | 127 | 109 | 50 | 21.2 | 1/50 | 2.0 | 23/65 | 35.4 | 12/51 | 23.5 | 14/70 | 20.0 |
| 3 - 4 | 283 | 155 | 128 | 30 | 10.6 | 5/73 | 6.85 | 12/74 | 16.2 | 6/72 | 8.33 | 7/64 | 10.9 |
| 5 – 6 | 268 | 159 | 109 | 21 | 7.84 | 5/71 | 7.04 | 6/95 | 6.32 | 4/65 | 6.15 | 6/37 | 16.2 |
| 7 – 8 | 250 | 130 | 120 | 21 | 8.4 | 4/72 | 5.56 | 10/86 | 11.6 | 3/56 | 5.36 | 4/36 | 11.1 |
| 9 – 10 | 227 | 114 | 113 | 22 | 9.69 | 5/71 | 7.04 | 4/78 | 5.13 | 7/45 | 15.6 | 6/33 | 18.2 |
| 11 – 12 | 146 | 77 | 69 | 13 | 8.9 | 1/40 | 2.5 | 7/41 | 17.1 | 2/37 | 5.41 | 3/28 | 10.7 |
| 13 – 14 | 147 | 75 | 72 | 10 | 6.8 | 0/38 | 0 | 7/41 | 17.1 | 1/35 | 2.86 | 2/33 | 6.06 |
| 15 – 20 | 452 | 171 | 281 | 37 | 8.19 | 7/113 | 6.19 | 6/119 | 5.04 | 4/97 | 4.12 | 20/123 | 16.3 |
| 21 – 30 | 493 | 168 | 325 | 86 | 17.4 | 11/133 | 8.27 | 11/108 | 10.2 | 9/109 | 8.26 | 55/143 | 38.5 |
| 31 – 40 | 592 | 170 | 422 | 261 | 44.1 | 56/149 | 37.6 | 56/127 | 44.1 | 30/135 | 22.2 | 119/181 | 65.8 |
| 41 – 50 | 525 | 188 | 337 | 398 | 75.8 | 109/145 | 75.2 | 110/130 | 84.6 | 75/119 | 63.0 | 104/131 | 79.4 |
| > 50 | 549 | 199 | 350 | 508 | 92.5 | 139/147 | 94.6 | 121/125 | 96.8 | 108/120 | 90.0 | 140/157 | 89.2 |
| Total | 4,260 | 1,792 | 2,468 | 1,471 | 34.53 | 346/1,125 | 30.8 | 375/1,109 | 33.8 | 262/961 | 27.3 | 488/1065 | 45.8 |
